# Exposure to political violence and health risk behaviors of Palestinian youth

**DOI:** 10.1186/s12889-025-23191-4

**Published:** 2025-06-02

**Authors:** Rita T. Karam, Wenjing Huang, Umaiyeh Khammash, Peter Glick, Mohammed Shaheen, Ryan Andrew Brown, Sebastian Linnemayr, Salwa Massad

**Affiliations:** 1 RAND Corporation, 1776 Main Street, Santa Monica, CA USA; 2 Juzoor for Health and Social Development, Ramallah, Palestine; 3 Millenium Challenge Corporation, 1099 14 th Street NW, Suite 700, Washington, D.C. 20005 USA; 4 Daralkalima University, Bethlehem, Palestine; 5 The World Health Organization, Jerusalem, Palestine

**Keywords:** Palestinian youth, Political violence, Risky behavior, Adolescent health, Mental health, Drug use, Alcohol use, Sexual activity

## Abstract

**Background:**

Exposure to political violence, which pervades many parts of the Middle East and Northern African (MENA) region, is a key potential factor behind the rising rates of risky behaviors among youth, such as drug use, alcohol use, and sexual activity. Theory and empirical work on youth elsewhere suggests that individual characteristics, mental health, and youths’ future orientation play a role in such behaviors. It is possible that political violence impacts behavior in part through its effects on these factors, in particular mental health. However, very little is known about the determinants of youth risk behavior in the region. Understanding the determinants will help MENA countries to deal with emerging public health threats as well as risks to youth health and well-being resulting from engagement in risky behavior. We examined determinants of risky behavior among Palestinian youth in the West Bank and East Jerusalem.

**Methods:**

We employed structural equation modeling using a 2014 nationally representative data from the Palestinian Youth Health Risk Study to examine the factors associated with engagement of youth ages 18–24 (*N* = 1449) in risky behaviors.

**Results:**

Personal experience of political violence was the strongest direct predictor of engagement in interpersonal violence (*β* = 0.21,* p* = 0.00) and substance use (*β* = 0.21,* p* = 0.00). With respect to indirect effects, global distress mediates the impact of witnessing and vicariously experiencing violence on the three outcomes. However, no association was found between personally experiencing political violence and global distress. The study also identified several individual characteristics, such as religiosity, that may be protective against risky behavior. Females are less likely to engage in risky behavior than males, despite experiencing higher levels of global distress.

**Conclusions:**

The study is the first to use population-based data to test the effects of exposure to political violence on key risky health behaviors of Palestinian youth, a population facing protracted conflict and hardship for which solutions remain elusive. The findings suggest the need for customized interventions to target male and female Palestinians at an early age to develop their coping skills in dealing with violence and distress.

**Trial registration:**

Not applicable.

## Background

Political violence can create an environment that increases risky behaviors among youth such as drug use and violence. Many countries in the Middle East and Northern African (MENA) region, which are experiencing political violence, are also exhibiting high rates of risky behaviors among youth, including tobacco and drug use, sexual activity, and violence [1, 2]. Illicit drug use among youth has emerged as a serious public health issue in several MENA countries [3–5]. Similarly, studies have found increases in sexually transmitted infections among youth, indicating that rates of pre-marital sexual activity are rising in the region [1]. Further, violent behavior has been found to be highly prevalent in countries in MENA affected by armed conflict [2].

There is growing concern about the increased risk behaviors among youth in the MENA region because it can lead to severe consequences that affect not only the individual but also the community at large via, for example, the spread of infectious diseases, strains on the health care system, increased family conflict, and lost productivity [3]. The Palestinian Youth Health Risk Study, based on a nationally representative household survey of youth aged 15–24 years in the West Bank including East Jerusalem (also referred to as the Occupied Palestinian Territories or oPt), found that Palestinian youth are experiencing similar levels of risky behavior to those in other parts of the MENA region [6].

While there is growing concern over risky behaviors among youth in the MENA region, very little is known about the determinants of such behavior in the region. A key potential factor is exposure to political violence, which pervades many parts of the region. Theory and empirical work on youth elsewhere also suggests that individual characteristics such as mental health and youths’ future orientation or sense of control (which may themselves be affected by violence) may also play a role. Understanding such determinants will help MENA countries to deal with emerging public health threats as well as risks to youth health and well-being resulting from engagement in risky behavior.

The present study uses 2014 data from the Palestinian Youth Health Risk Study to examine the factors associated with youth engagement in risky behaviors. Unusual for research addressing such behaviors of youth in the region, the study collected large-scale, representative data both on a range of risky behaviors (smoking, drug and alcohol use, pre-marital sex, and violent behavior) and on many potential determinants or mediators of these behaviors [6]. We use these unique data to consider the effect of exposure to political violence on risky behaviors of Palestinian youth, including the role of several potential mediating factors in this relationship suggested by prior literature on youth: global distress (mental distress), fatalism, and impulsivity.

### Literature

Youth in oPt are profoundly affected by the Israeli-Palestinian conflict and the Israeli occupation [7, 8]. However, there is dearth of research examining how political violence affects youth’s risky behavior within oPt. Research in other settings points to associations between experiencing political violence and behavioral problems among youth, such as bullying, fighting, and antisocial behavior [9, 10]. The literature also indicates that the connection between political violence and risky behavior is complex and includes indirect paths through mental health and other well-being related factors. Exposure to political violence is associated with a range of mental health problems among young populations, including Palestinian youth [7, 8]. Specifically, exposure to political violence may cause depression and anxiety, referred to as global distress, that compromises cognitive function, which in other contexts has been shown to lead to engagement in high-risk activities to cope with untenable situations [7, 11, 12]. Youth struggling with depression and anxiety will seek ways to cope with their emotional distress, that can sometimes manifest in in unhealthy ways such as substance abuse and unsafe sexual activities as means to escape their feelings. Theoretical models of health and risk behaviors also consider how an individual’s view of how the world works, such as a fatalistic orientation (possibly affected by experience of violence or conflict), affects behavior and health outcomes [13, 14]. While earlier research considered fatalistic orientation as an enduring trait, other research has found that the degree of fatalism is affected by the context and circumstances under which people live [15, 16]. This more recent work suggests that environments like the oPt that are marked by violent conflict and uncertainty may promote fatalistic attitudes among youth, in turn leading to increased engagement in risky behaviors. Youth who view their fate to be unalterable may perceive risky behavior as less dangerous since such actions are not likely to impact their future one way or the other. They may also lack motivation to engage in behaviors that promote future success and focus on behaviors that provide instant gratification such as substance abuse.

Research also suggests that exposure to violence compromises key self-regulatory functions such as impulse control, thus increasing the likelihood of engaging in risky behavior [17, 18]. Specifically, young people who are exposed to violence may experience decreased neural connectivity in areas of the brain responsible for emotion regulation and cognitive control, thus leading to difficulties in managing impulses, emotions, and behaviors [19]. Greater impulsivity is likely to cause individuals to react to events or stimuli immediately rather than processing the information thoroughly, hence with less regard to possible negative consequences [20, 21]. The literature suggests individuals with low levels of self-control are more likely to engage in criminal activity or risky behaviors [22].

**Fig. 1:**
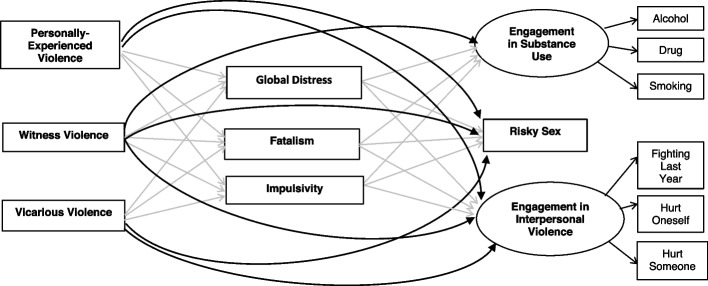
The structural equation model tested according to theory with all direct and indirect relationships estimated. Notes: 1. The effects of the control variables (e.g., age and gender) on the outcomes and on the mediators are not plotted in this path diagram. These estimated coefficients are shown in Table 1. 2. The direct effects are plotted using thick black arrows. The indirect effects are indicated by thinner gray arrows that go from the predictors to the three mediators and then to the outcomes

Based on the literature we developed a theoretical model, shown in Fig. 1, that is tested within the oPt context.

Our analysis also includes other determinants that may be associated with risky behaviors. For example, descriptive analysis of quantitative and qualitative data from the Palestinian Youth Health Risk Study found higher rates of sexual activity, drug use, alcohol use, and violence among male youth than among females [6, 23]. Cigarette smoking and sexual activity of unmarried young men increased with age, while violent behavior decreased with age. Previous studies in this population and others found dispossession and livelihood restriction are significant contribution factors to risky behaviors and outcomes [24, 25]. Further, studies found youth living in urban areas and refugee camps were significantly more likely than rural youth to engage in risky behaviors [6, 26]. Studies have also linked higher levels of religiosity with increased levels of depression and anxiety in adolescents and have showed that having a family member killed is a consistently significant predictor of post-traumatic stress disorder (PTSD) among adolescents [26].

The present study extends this literature in several ways. It is one of the few for the MENA region, and the first for the oPt, to examine the role of exposure to political violence and related factors in shaping youth health risk behaviors; the bulk of research on the region to date has focused on outcomes such as PTSD and other psychiatric symptomology. Even rarer are studies that, like this one, use large scale representative survey data, especially data including out-of-school and older youth who are likely at greater risk for destructive behaviors; most surveys of youth in prior research used school-based samples. The uniqueness of this data used in the study sheds new light on how factors such as mental health, fatalism, and impulse control mediate the relationship between violence exposure and risky behaviors in a highly vulnerable youth population.

## Methods

### Data

The Palestinian Youth Health Risk Study is a 2014 national household survey on the prevalence of health risk behaviors among Palestinian youth aged 15 to 24 in the West Bank including East Jerusalem. Detailed information on sample recruitment and data collection procedures can be found at https://journals.plos.org/plosone/article?id=10.1371/journal.pone.0198435. In this paper we use a subsample (*N* = 1,449) of older youth 18–24, since questions on sexual behaviors were not asked of younger respondents due to the cultural sensitivity of the topic. Youth consent/assent and (for minors) parental consent was obtained for the interviews. The study was approved by RAND’s Institutional Review Board.

### Measures

The measures are below grouped by predictors, mediators, and outcomes. See Glick et al., 2018 for more detailed information on measures.

### Predictors

#### Exposure to political violence

Exposure to political violence was measured by asking respondents if they had ever experienced any of a list of 11 events categorized under three types of violence exposure. We differentiate between the three types of violence exposure because research suggests they may have distinct psychological or behavior impact on individuals. For example, individuals who personally experience political violence may experience more severe trauma and psychological effects than those who hear about such violence [27].

*Personally experienced political violence* (4 events) was measured by whether individuals reported being physically assaulted by soldiers or police; shot by rubber/real bullets; imprisoned or held by police/other authority; or had house or family home closed/demolished by Israelis or others.

*Witnessed political violence* (4 events) was measured by whether individuals reported directly witnessed beating of close relative/friend; directly witnessed killing of close relative/friend; witnessed shooting of close relative/friend by rubber/real bullets; and directly witnessed a close relative’s/friend's/neighbor’s house closure or demolition.

*Vicarious* (or heard about) political violence (2 events) was measured by whether individuals reported having a close relative/friend who was killed, though they did not witness it; and had a close relative who was imprisoned or held.

Each item elicited a yes (1)/no (0) response. For each type of violence, the responses for the dichotomous variables that represent whether any events of that type had been experienced were summarized and averaged so that the mean is bounded between 0 and 1. These events largely refer to actions carried out by authorities, and are likely to consist mainly, though not necessarily exclusively, of actions by Israeli forces (some may be attributable to the Palestinian Authority police; the survey purposively did not distinguish the source, to encourage greater response rates).

### Mediators

#### Global distress (Mental Health)

Global distress was measured using the 25-item Hopkins Symptoms Checklist (HSCL-25) [28]. HSCL-25 measures symptoms of anxiety (10 items) and depression (15 items). Each item asked for whether the symptoms bothered or distressed the participant in the last week. Examples of items include: (1) feeling lonely, (2) feeling slow in energy, (3) thoughts of ending your life, and (4) suddenly scared for no reason. Response options for each item range from 1 ‘not at all’ to 4 ‘extremely’. Cronbach’s alpha was 0.91 indicating high scale reliability. Responses were averaged to generate a score for global distress. A cut-off greater than 1.75 represent elevated global distress [7, 29].

#### Fatalism

We selected 3 items each from two 6-item subscales of the multidimensional fatalism scale developed by Esparza et al., [30]. The scale was reduced to keep the overall survey to a practical length. Participants were asked to indicate their level of agreement (1 ‘strongly agree’ to 5 ‘strongly disagree’) with statements on God and individual control of life events. Examples of statements include: (1) what happens to me in the future mostly depends on me; (2) whatever happens is because of God; and (3) what people get out of life is always due to effort.

We scored fatalism using a latent variable approach based on the bi-factor model to correct for measurement errors and local dependency [31]. The approach demonstrated very good model fit (RMSEA = 0.04). The general factor scores were calculated using IRTPRO [32] and were treated as a single observed variable with higher scores representing greater fatalism.

#### Impulsivity

We used the reduced Barratt Impulsiveness Scale-Brief (BIS-Brief) [33] to assess general impulsiveness. The shorter scale includes 8 items. Examples of the items include: (1) I say or do things without thinking; (2) I act on the spur of the moment; and (3) I do not pay attention to what I do. The responses are on a scale of 1 to 4 where 1 = rarely/never, 2 = occasionally, 3 = often, and 4 = almost always/always. We scored the scale by taking the average of the 8 items. Cronbach’s alpha was 0.58 indicating low internal reliability.

### Risky behavior outcomes

#### Substance use

We used three dichotomous indicators to measure substance use as a latent variable including: 1) ever used alcohol; 2) ever used any illicit drug; and 3) currently smokes.

#### Risky sexual behavior

Risky sexual behavior was defined by whether the participant had premarital sexual intercourse. It is considered a risk behavior from a health perspective given the possibility of sexually transmitted infection. There is a lack of appropriate education in the oPt about engaging in safe sexual activity that can lead to health risks. Further, in the oPt context, premarital sex is socially risky as it would be viewed by many as a violation of cultural norms. Risky sexual behavior is treated as an observed dichotomous outcome variable in the model. For married respondents, having engaged in premarital sexual intercourse is inferred from responses for age at marriage and age at first intercourse.

#### Engagement in interpersonal violence

This includes three questions: 1) whether one engaged in a physical fight with someone last year; 2) had ever been hurt or injured in a fight as a youth or an adult; or 3) were ever hurt or injured someone else in a fight as youth or an adult. Since the response categories of these three aggressive behaviors were on different-ordered categorical scales (question 1 is on a 5-point scale, while questions 2 and 3 are on a 4-point scale), we used them as separate indicators in the model to measure ‘engagement in interpersonal violence’ as a latent factor. It should be noted that these measures of interpersonal violence are conceptually distinct from the exposure to violence indicators, which refer to actions carried out by the authorities or related to the conflict.

Other determinants include age, sex, ever married, an asset index constructed from data on consumer durables (see Glick et al., 2018), years of completed schooling, religiosity (based on responses to ‘Do you consider yourself religious?’ (Yes–very, Yes–sometimes, No)), residential location (urban, rural, or refugee camp), whether the respondent experienced mother or father death and if father is living, whether he is experiencing economic hardship (is unemployed or disabled/chronically ill). For religiosity we recoded the scale into Yes (for ‘Yes—very’ or ‘Yes—sometimes’) and No.

### Statistical analyses

Structural equation modeling (SEM) was used to assess the relationships between the abovementioned constructs. We estimated SEM using Mplus 8 with the weighted least squares estimation with missing data (WLSMV) method. For each estimated regression parameter, we report its standardized estimate (β), its standard error (SE), and the *p*-value. To evaluate model fit, we report the Root Mean Square Error of Approximation [34], Comparative Fit Index [35], and the Standardized Root Mean Square Residual (SRMR). Satisfactory global model fit is attained when two of the following three conditions are met: CFI ≥ 0.95, RMSEA ≤ 0.06, and SRMR ≤ 0.08 [36].

## Results

### Sample description

The sample has a mean age of 20.8 years (SD = 2.02), with 52% being females and 14.6% being married or ever married. About 26.8% have fewer than 12 years of education, among which 8.6% are still in school. The majority reside in urban areas (66.2%); 7.9% are from refugee camps, and the rest are from rural areas. A large majority reported being either somewhat religious (65.2%) or very religious (17.8%). The majority (85.6%) experienced one or more violent events (direct, witnessed, or vicarious) in their lifetime. Looking separately by type of event, 27.5% had been a victim of a personal violent act; 72.3% had witnessed at least a violent act perpetrated on a relative or close friend, and 72.1% had heard of a violent act experienced by a relative or close friend.

More than half of the sample (54.9%) had elevated symptoms of global distress. Most participants seemed to have a fatalistic orientation when asked about the role of God, with 65% agreeing, for example, that ‘whatever happens is because of God’. At the same time, they also tended to see themselves as the locus of control. For example, 55% agreed that ‘what happens to me in the future mostly depends on me’. More than 50% of participants reported at least occasionally engaging in an impulsive behavior.

Regarding engagement in risky behaviors, 49.1% of the sample reported currently smoking (cigarettes or waterpipe). About 68% of males and 30.9% of females were current smokers. Experience with alcohol and drugs is much lower but not insignificant, with 14.8% reported having tried alcohol and 6.3% reported having tried any drug. Loadings of smoking, drinking, and drug indicators on the latent variable ‘substance use’ is high (0.86, 0.76 and 0.75 respectively), indicating that the items effectively reflect and measure ‘engaging in risky behavior’.

The prevalence of risky sex was higher among the unmarried than among the married (the latter inferred from reported ages at marriage and first intercourse, as noted). Among the unmarried, 7.2% were at risk. Among those that were ever married, 4.9% reported having had sexual intercourse before marriage.

Rates of engagement in interpersonal violence (fighting) were high. Approximately 43% of male and 21% of female youth engaged in a physical fight with someone at least once in the year prior to the survey and 29% of male and 13% female got hurt or injured in a fight. In addition, 39% male and 11% female hurt or injured someone else in a fight. These three indicators load positively on the engagement of interpersonal violence latent factor with loadings of 0.65, 0.62 and 0.75 respectively. These loadings are moderate, suggesting the items have an adequate fit with the interpersonal violence latent construct.

### Results of SEM

**Fig. 2:**
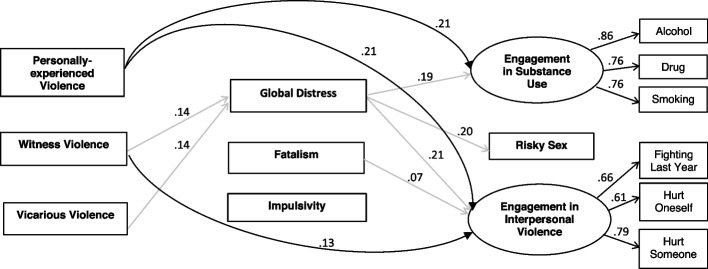
Structural Equation Model with statistically significant regression paths. Note: Statistically non-significant relationships are omitted from the figure

The SEM estimates indicate that the data fit the hypothesized structural equation model well (RMSEA = 0.03 well-bounded with 90% CI of 0.03 and 0.04; CFI = 0.95; TLI = 0.89). Figure 2 shows only the statistically significant paths (i.e., *p* value < 0.05) in the final model. The black lines with corresponding standardized regression coefficients represent the direct effects of the predictors on the outcomes; the grey lines represent indirect effects via the mediators.

Overall, we found statistically significant total effects of personally experienced political violence on all behavior three outcomes. The direct effects were much larger than the indirect effects. Table 1 below presents the standardized coefficients and *p*-values of the total effects, direct effects, and indirect effects.

**Table 1:** Standardized effects of violence exposure on risky behaviors, by direct and indirect effects

	On substance use		On risky sex		On engagement in interpersonal violence	
Personal experienced violence	β (s.e.)	*p* value	β (s.e.)	*p* value	β (s.e.)	*p* value
Total effect	.21 (.03)	0	.11 (.05)	.05	.22 (.03)	0
Direct effect	.21 (.03)	0	.10 (.05)	.07	.21 (.03)	0
Total indirect effects	.01(.006)	.13	.01 (.007)	.17	.01 (.007)	.13
Specific pathways through						
* A: global distress*	.01 (.006)	.09	.01 (.006)	.09	.01 (.007)	.09
* B: fatalism*	0 (.002)	.87	0 (.001)	.87	0 (.002)	.87
* C: impulsivity*	-.001 (.001)	.54	-.001 (.002)	.59	-.001 (.001)	.42
Witnessed violence	β (s.e.)	*p* value	β (s.e.)	*p* value	β (s.e.)	*p* value
Total effect	.07 (.04)	.06	.06 (.06)	.28	.16 (.03)	0
Direct effect	.04 (.04)	.23	.03 (.06)	.57	.13 (.03)	0
Total indirect effects	.02 (.007)	.001	.02 (.007)	.003	.03 (.007)	0
Specific pathways through						
* A: global distress*	.03 (.007)	0	.03 (.008)	.00	.03 (.007)	0
* B: fatalism*	-.002 (.002)	.33	.001 (.002)	.50	-.002 (.002)	.30
* C: impulsivity*	-.001 (.001)	.58	-.001 (.002)	.62	-.001 (.001)	.50
Vicarious or heard about violence	β (s.e.)	*p* value	β (s.e.)	*p* value	β (s.e.)	*p* value
Total effect	.09 (.04)	.01	.02 (.06)	.75	.09 (.03)	.01
Direct effect	.06 (.03)	.08	-.01 (.06)	.84	.06 (.03)	.06
Total indirect effects	.03 (.007)	0	.03 (.008)	.00	.03 (.007)	0
Specific pathways through						
* A: global distress*	.03 (.006)	0	.03 (.008)	.00	.03 (.007)	0
* B: fatalism*	-.001 (.002)	.68	.00 (.001)	.71	-.001 (.002)	.68
* C: impulsivity*	0 (.001)	.80	.00 (.001)	.80	0 (.001)	.79

Personally experienced political violence was the strongest direct predictor of two of the risky behavior outcomes: engagement in interpersonal violence (*β* = 0.21,* p* = 0.00) and substance use (*β* = 0.21,* p* = 0.00). Witnessing political violence toward relatives or close friends had a (very small) direct impact only on engagement in interpersonal violence (*β* = 0.13,* p* = 0.00). Hearing about violence affecting relatives or close friends did not contribute directly to any of the risky behavior outcomes. In contrast to their effects on substance use and interpersonal violence, none of the political violence measures have direct effects on risky sexual behavior.

With respect to indirect effects, there is only limited evidence for mediation of the effects of the three types of exposures to political violence. Global distress, but not the other two hypothesized mediators (fatalism or impulsivity) mediate the impact of witnessing and vicariously experiencing violence on the three outcomes. These indirect effects were very small compared to direct effects exposure to violence on the outcomes.

We did not find any association between personally experiencing political violence and global distress.

### Effects of the other determinants

Our analysis also showed that certain individual characteristics and contexts had sizable effect sizes in predicting outcomes (Table 2).

**Table 2:** Effects of the control variables on the mediators and on the outcomes

	***Outcomes***			***Mediators***		
	Substance use	Risky sex behaviors	Engagement in interpersonal violence	Global distress	Fatalism	Impulsivity
	β (s.e.)	β (s.e.)	β (s.e.)	β (s.e.)	β (s.e.)	β (s.e.)
Age	0.17 (.04)*	0.16 (.06)*	−0.05 (.03)	0 (.03)	0 (.03)	0.12 (.03)*
Gender	−0.29 (.04)*	−0.02 (.06)	−0.29 (.04)*	0.36 (.03)*	−0.12 (.03)*	−0.04 (.03)
Ever married	−0.08 (.04)*	−0.07 (.07)	0 (.04)	−0.05 (.03)	−0.06 (.03)*	−0.04 (.03)
Years of education	−0.15 (.04)*	−0.14 (.06)*	−0.08 (.03)*	−0.06 (.03)*	−0.01 (.03)	0.03 (.03)
Asset	0.19 (.03)*	0.15 (.05)*	0.07 (.03)*	−0.01 (.03)	0.05 (.03)	0.08 (.03)*
Religiosity	−0.31 (.03)*	−0.37 (.05)*	−0.04 (.03)	−0.07 (.03)*	0.05 (.03)	0.06 (.03)*
Father death	0.03 (.03)	0 (.05)	−0.01 (.03)	0.06 (.02)*	−0.02 (.03)	−0.03 (.03)
Mother death	0.07 (.03)*	0.04 (.04)	−0.01 (.03)	0.05 (.02)*	−0.02 (.02)	−0.01 (.02)
Father hardship	−0.05 (.03)	−0.01 (.05)	−0.03 (.03)	0.01 (.02)	−0.01 (.03)	0.03 (.03)
Urban areas	0.24 (.03)*	0.22 (.07)*	0.08 (.03)*	−0.04 (.03)	0.05 (.03)	0.03 (.03)
Refugee camps	0.13 (.03)*	0.07 (.06)	0.07 (.03)*	−0.06 (.03)*	0 (.03)	0.03 (.03)

*Are statistically significant

The level of religiosity was found to be one of the strongest predictors of risky behavior: increased religiosity was associated with lower engagement in risky sexual behavior (*β* = −0.37, *p* < *0.05*) and substance use (*β* = −0.31, *p* < *0.05*). There was no relationship between religiosity and engagement in interpersonal violence. Females reported less substance use (*β* = −0.29, *p* < *0.05*) and less engagement in interpersonal violence (*β* = −0.29,* p* = < 0.05) than males. Being female was not associated with lower engagement in risky sexual behavior. Being female is also associated with increased global distress (*β* = 0.36,* p* < 0.05), and with less fatalistic views of life events (*β* = −0.12,* p* < 0.05).

Residential location was also found to be a strong predictor of risky behavior. Youth living in urban areas were more likely to engage in substance use (*β* = 0.24, *p* < *0.05*), interpersonal violence (*β* = 0.08, *p* < *0.05*), and risky sexual behavior (*β* = 0.22, *p* < *0.05*) than youth in rural areas. Similarly, youth with higher education levels tended to engage less in each risky behavior and reported lower global distress. Other individual characteristics such as marital status, family assets, and parent death were found to have very small impacts on very few behavioral outcomes and mediators.

## Discussion

The study is the first to use population-based data to test the effects of exposure to political violence on key risky health behaviors of Palestinian youth, a population facing protracted conflict and hardship. We theorized that the three types of political violence experienced (personal, witnessed, and vicarious) had both direct and indirect effects on these behaviors (substance use, risky sex, and engagement in interpersonal violence), with indirect effects working through mental health, fatalism, and impulsivity. Our analysis found that personal experience of political violence had the largest direct impact on youths’ engagement in substance use and in aggressive acts. This is consistent with studies of youth exposure to community violence in other regions [37]. In contrast, witnessing or hearing about political violence affecting close friends/relatives had direct effects only on interpersonal violence.

We also found that witnessing and hearing about violence had a small indirect effect via global distress on the three risky behaviors. No such mediated effects were found, however, for personal exposure to political violence.

While witnessing and hearing of political violence were independently associated with increased (worse) global distress, surprisingly personal exposure to political violence was not. This is inconsistent with other studies of youth exposure to community violence that were conducted in and outside conflict settings including oPt [38]. A possible explanation is that the strength of the direct effects of personally experiencing political violence on risky behavior has overshadowed any association it may have through mental health. Another possible explanation is while hearing of or witnessing the suffering of others can impact anxiety and depression, personal exposure to traumatic events might lead to other mental health conditions such as PTSD that is not measured in our study.

In contrast to other studies and our hypothesis, we also found that within the Palestinian context, impacts of personal exposure to political violence on risky behaviors were not mediated through youth’s fatalistic views and impulsive behaviors. In fact, personal exposure to political violence did not seem to have any direct effects on these two mediating variables. A caveat, however, is that, as mentioned above, both constructs had low reliability, which may affect the results.

Our analysis also identified individual characteristics that have direct impacts on risky behavior. Male youth engaged more in risky behaviors than did female youth. Such gender differences could be explained by the conservative and patriarchal nature of Palestinian society. Relative to their male counterparts, female youth face family or community restrictions on their mobility and participation in social activities that might encourage risky behavior. On the other hand, female youth had elevated symptoms of global distress compared to males, as reported above and in a previous study [7]. It is plausible that poorer mental health among young women reflects these same gender-specific constraints. We also found youth who reported high level of religiosity engaged less in substance use and sexual behavior outside marriage. This suggests that religiosity may be protective against such behaviors, though it may also reflect unmeasured individual traits associated both with devotion to Islam and (low) engagement in the behaviors.

### Implications

The study shows that personal exposure to political violence—an individual’s direct victimization from such violence, including that related to occupation and the prolonged Israeli-Palestinian conflict—increases vulnerability of Palestinian youths to a range of health risk behaviors. Since the start of the Israel-Hamas conflict in 2023, there has been a significant increase in political violence in the West Bank and East Jerusalem by Israeli settlers and heightened military operations against Palestinians. This includes violent attacks on Palestinians causing injury and death, and the destruction and confiscation of property. This escalation can be expected to have additional harmful impacts on Palestinian youths’ mental health, risk taking behavior, and social interactions. There is of course, the urgent need to end the conflict and occupation which are sources of much of the violence youth in the oPt experience. Beyond this (at best) long term objective, our findings provide some guidance regarding prevention programs. Male Palestinian youth who experienced personal violence appear to be particularly at risk for engaging in risky behavior. While there are prevention programs provided by international organizations and local communities in oPt to support individuals experiencing political violence, there is stigma for men to seek and utilize such support due to cultural norms. This suggests that efforts need to be made to destigmatize such services for men by launching education campaigns through social media, community events, as well as religious institutions. The campaigns could encourage male role models, influencers, and public figures to speak openly about their struggles to normalize the conversation about mental health and risky behavior and improve uptake of available services. Further, given that Palestinian women have higher levels of global distress than males, it becomes also important that other interventions are tailored specifically to address female depression and anxiety symptoms. These interventions would also need to be designed carefully to overcome any stigma related to mental health and to encourage participation.

Having adequate prevention and mental health programs in place is challenging in oPt due to limited resources and restricted movement for both those in need of support and healthcare providers. However, there are strategies that could be taken to overcome some of the challenges. Technology can be used to provide counseling and other support services to reach those who face movement restrictions or who are in remote areas. This can also make it easier for individuals to seek help privately and without fear of judgment. The education system can also have a role in overcoming challenges for implementing prevention programs. The fact that school enrollment in oPt is very high at the primary grade level provides an opportunity to incorporate prevention program that address the development of coping mechanisms and social and emotional learning as part of the school curriculum. This will help individuals at an early age develop the skills they need to manage their emotions and stress as a result of political violence in order to make sound decisions.

Our study has limitations that point to the need for additional research. First, the study is cross- sectional, which makes interpretation ambiguous since relationships may be bi-directional. For example, global distress may lead to risky behavior, but the latter may also contribute to global distress. Longitudinal research is therefore needed to best capture these relationships. Second, the study relies on self-reports of risky behavior and mental health symptoms rather than, for example, clinical data to assess mental health status. Social desirability bias may have influenced participant responses, particularly regarding mediating and risk behavior outcome variables. There is possibly also a degree of recall bias since some of the questions require respondent to remember past events. Finally, while our model achieved a good fit, other unmeasured, factors may contribute to both the mediators and outcomes—e.g., parenting practices, availability of support services, family and peer support, and individual resilience characteristics—clouding a causal interpretation. Even with these limitations, however, the unique representative data used in this study has provided important insights on prevalence of health risk behaviors among Palestinian youth and the factors influencing such behaviors.

## Conclusions

The paper provides novel findings as it is the first to use population-based data to test the effects of exposure to political violence on key risky health behaviors of Palestinian youth. Our analysis found that individual’s direct experience of political violence, including that associated with the occupation and prolonged Israeli-Palestinian conflict, had the largest direct impact on youths’ engagement in substance use and in aggressive acts. Witnessing and hearing of political violence increased mental health issues (global distress) and had an indirect effect via global distress on youth’s engagement in substance use, aggression, and risky sexual behavior. Male reported more substance use and engagement in aggressive behavior than women. However, women exhibited elevated levels global of distress compared to men possibly because of cultural constraints they experience. The findings imply the need for early interventions that consider cultural values and gender to promote Palestinian youth protective factors from an early age, and careful design of youth interventions to deal with challenges of both stigma and constraints imposed by the occupation.

## Data Availability

Data is provided within the manuscript.

## Abbreviations

BIS: Barratt Impulsiveness Scale-Brief
MENA: Middle East and Northern African
oPt: Occupied Palestinian Territories
PTSD: Post-traumatic Stress Disorder
